# Prevalence and associated factors of oligohydramnios in pregnancies beyond 36 weeks of gestation at a tertiary hospital in southwestern Uganda

**DOI:** 10.1186/s12884-022-04939-x

**Published:** 2022-08-02

**Authors:** Godfrey Twesigomwe, Richard Migisha, David Collins Agaba, Asiphas Owaraganise, Hillary Aheisibwe, Leevan Tibaijuka, Lenard Abesiga, Joseph Ngonzi, Yarine Fajardo Tornes

**Affiliations:** 1 Department of Obstetrics and Gynecology, Mbarara University of Science and Technology, P.O Box 1410, Mbarara, Uganda; 2 Department of Physiology, Mbarara University of Science and Technology, P.O Box 1410, Mbarara, Uganda

**Keywords:** Oligohydramnios, Amniotic fluid volume, Prevalence, Amniotic fluid index, Uganda

## Abstract

**Background:**

Oligohydramnios is associated with poor maternal and perinatal outcomes. In low-resource countries, including Uganda, oligohydramnios is under-detected due to the scarcity of ultrasonographic services. We determined the prevalence and associated factors of oligohydramnios among women with pregnancies beyond 36 weeks of gestation at Mbarara Regional Referral Hospital (MRRH) in Southwestern Uganda.

**Methods:**

We conducted a hospital-based cross-sectional study from November 2019 to March 2020. Included were women at gestational age > 36 weeks. Excluded were women with ruptured membranes, those in active labour, and those with multiple pregnancies. An interviewer-administered structured questionnaire was used to capture demographic, obstetric, and clinical characteristics of the study participants. We determined oligohydramnios using an amniotic fluid index (AFI) obtained using an ultrasound scan. Oligohydramnios was diagnosed in participants with AFI ≤ 5 cm. We performed multivariable logistic regression to determine factors associated with oligohydramnios.

**Results:**

We enrolled 426 women with a mean age of 27 (SD ± 5.3) years. Of the 426 participants, 40 had oligohydramnios, for a prevalence of 9.4% (95%CI: 6.8–12.6%). Factors found to be significantly associated with oligohydramnios were history of malaria in pregnancy (aOR = 4.6; 95%CI: 1.5–14, *P* = 0.008), primegravidity (aOR = 3.7; 95%CI: 1.6–6.7, *P* = 0.002) and increasing gestational age; compared to women at 37–39 weeks, those at 40–41 weeks (aOR = 2.5; 95%CI: 1.1–5.6, *P* = 0.022), and those at > 41 weeks (aOR = 6.0; 95%CI: 2.3–16, *P* = 0.001) were more likely to have oligohydramnios.

**Conclusion:**

Oligohydramnios was detected in approximately one out of every ten women seeking care at MRRH, and it was more common among primigravidae, those with a history of malaria in pregnancy, and those with post-term pregnancies. We recommend increased surveillance for oligohydramnios in the third trimester, especially among prime gravidas, those with history of malaria in pregnancy, and those with post-term pregnancies, in order to enable prompt detection of this complication and plan timely interventions. Future longitudinal studies are needed to assess clinical outcomes in women with oligohydramnios in our setting.

## Introduction

Oligohydramnios is the commonest amniotic fluid disorder, characterized by reduced amniotic fluid or amniotic fluid volume that is less than expected for gestational age [1]. Oligohydramnios is associated with poor maternal and fetal/neonatal outcomes, including intrauterine growth restriction, fetal distress, birth asphyxia, prolonged labor, and increased risk of caesarean section, often arising from umbilical cord compression, meconium aspiration, or uteroplacental insufficiency [2, 3]. In low-and middle-income countries, oligohydramnios accounts for approximately up to 6.5% of still births [4].

Oligohydramnios occurs in about 1–5% of term pregnancies worldwide; however, the prevalence rises to more than 12% in post-term pregnancies [5–7]. In Africa, prevalence rates of oligohydramnios ranging from 4 to 23% have been reported previously [8]. Additionally, several maternal, placental, and fetal factors, including ruptured amniotic membranes, fetal abnormalities, genetic factors, maternal illnesses, nutrition status, multiple pregnancies, use of non-steroidal anti-inflammatory drugs (NSAIDs), and use of certain angiotensin converting enzyme inhibitors (ACEIs), have been found to be associated with oligohydramnios [5].

Despite the poor perinatal outcomes attributable to oligohydramnios, there are few data on prevalence and factors associated with oligohydramnios in the East African Region, and Uganda in particular. Establishing the prevalence of oligohydramnios and associated factors in the third trimester is crucial to inform the index of suspicion among healthcare workers during antenatal care so as to plan appropriate interventions, including mode and timing of deliveries. In Uganda, many missed opportunities for prompt detection of oligohydramnios exist; only 30% of pregnant women requiring ultrasonography services access them [9, 10]. When oligohydramnios is missed and patients do not receive appropriate and timely treatment, outcomes are often poor.

Prior to conducting this study, data from review of medical records for a six-month period (April–September 2018) in the Department of Obstetrics and Gynecology at Mbarara Regional Referral Hospital (MRRH) revealed that 45 mothers had oligohydramnios-related complications. All the 45 mothers had cesarean sections, 6% had fresh stillbirths, and 17% had babies with birth asphyxia (APGAR scores of < 7 at 5 min). Furthermore, oligohydramnios may be contributing to the high rates of cesarean section deliveries; fetal distress caused by oligohydramnios has been identified as one of the leading indications for cesarean section deliveries in Uganda [11]. MRRH, in particular, has been found to have high cesarean section delivery rates of more than 25% [12, 13]. This study aimed to determine the prevalence and associated factors of oligohydramnios among women attending antenatal care at > 36 weeks of gestation at MRRH in Southwestern Uganda.

## Methods

### Study population and setting

We conducted this cross-sectional study at Mbarara Regional Referral Hospital (MRRH) Maternity Ward and Antenatal Clinic from November 16, 2019 to March 12, 2020. MRRH is found in Mbarara District, 286 km southwest of Kampala, the capital city of Uganda. It is a public hospital under the Ministry of Health (MoH). It serves 11 districts with an estimated population of about five million individuals. The hospital also serves as a teaching hospital for undergraduate and postgraduate students of Mbarara University of Science and Technology (MUST). The maternity ward handles about 10,000 deliveries per year. At the antenatal admissions’ area, an average of 40 pregnant women are attended to on a daily basis. The hospital also has antenatal clinic that attends to approximately 40 pregnant mothers per day.

We included mothers who sought care at MRRH at a gestation age of 37 weeks or beyond, and who consented to participate in the study. We excluded mothers who had ruptured membranes, those in active stage of labour, and those with multiple pregnancies.

### Study definitions and procedures

We administered a structured questionnaire to obtain socio-demographic, obstetric, and clinical characteristics of the study participants. Socio-demographic factors included age, occupation. marital status, and education level. Obstetric factors included a history of placenta previa, hypertension in pregnancy, gravidity, gestational age, and parity. Medical factors included a history of infections including malaria, and HIV. In addition, data on history of anemia during pregnancy, were obtained. Gestational age was determined based on first day of the last normal menstruation period (LNMP) or an ultrasound scan done in the first trimester if available [14]. Antenatal cards were reviewed to verify the socio-demographic and clinical data, including data on booking blood pressure, LNMP, and history of infections, hypertension, and anemia during the current pregnancy.

All eligible participants who consented to participate underwent sonographic assessment for amniotic fluid index (AFI), using an ultrasound scanner (Edan Instruments, Inc., Shenzhen, China; manufactured in 2018) with a 3.5 MHz curvilinear abdominal transducer. The sonographer (KL) was a certified professional with a degree in radiography. For each participant scanned, the sonographer reported the deepest amniotic fluid pool depth in each of the four uterine quadrants, summed to an AFI. Additionally, estimated fetal weight was reported using the Hadlock formula [15]. Our dependent variable, oligohydramnios, was defined as an AFI ≤ 5 cm [1].

### Sample size and sampling

For this study, we calculated a sample size of 426 participants using a single population proportion formula in consideration of 95% confidence level, 5% precision, and design effect of 1, 50% prevalence rate of oligohydramnios [16], and after consideration of a 10% non-response rate, using Epi Info (version 7.1.4.0, CDC, Atlanta, US). Participants were enrolled through consecutive sampling.

### Data management and analysis

We entered data in EpiData 3.1 software (EpiData, Odense, Denmark) and exported the data to STATA version 13 (StataCorp, College Station, Texas, USA) for all statistical analyses. The prevalence of oligohydramnios was determined as the proportion of participants with oligohydramnios. We categorized all study variables; the categorical variables were described (as frequencies, and percentages) and compared among those with oligohydramnios and those without oligohydramnios using Chi square or Fischer’s exact tests. Additionally, continuous, normally distributed variables (e.g., age, fetal weight, body mass index) were described as means with standard deviations. Univariable and multivariable logistic regression analyses were used to identify factors associated with oligohydramnios. Covariates significant (with *P* < 0.05) at univariable analysis were included in the multivariable model to identify independent factors significantly associated with oligohydramnios (*P* < 0.05). We adjusted the final model for maternal age, as a potential confounder.

## Results

### Characteristics of study participants

Socio-demographic, clinical and obstetric characteristics of the study participants are shown in Table 1. We enrolled 426 participants with a mean age of 26 (± 5.3) years; the age range was 17–44 years. Of the 426 participants, most were married (96%), aged 25 years or older (63%), and were at 37–39 weeks of gestation (54%); about half were prime gravidas (51%). The mean gestational age was 39 (± 1.5) weeks. About one in ten (11%) of the participants had HIV in pregnancy, and 11 (2.6%) had evidence of low-lying placenta. Compared to participants with no oligohydramnios, those with oligohydramnios were significantly more likely to be of higher gestational age (*P* < 0.001), lower gravidity (*P* = 0.004), with a history of malaria in pregnancy (*P* = 0.001), and lower BMI (*P* = 0.007); other characteristics were similar between the two groups (Table 1).

**Table 1:** Socio-demographic, clinical and obstetric characteristics of study participants by diagnosis of oligohydramnios at Mbarara Regional Referral Hospital, southwestern Uganda, November 2019–March 2020

**Characteristic**	**Overall**	**Oligohydramnios**		
	**(*N* = 426),** **n (%)**	**Yes (*n* = 40),** **n (%)**	**No (*n* = 386),** **n (%)**	***P* value**
**Age** ^a^ **category**				0.903
< 25 years	156 (36.6)	15 (37.5)	141 (36.5)	
≥ 25 years	270 (63.4)	25 (62.5)	245 (63.5)	
**Marital status**				0.613
Married	409 (96.0)	39 (97.5)	370 (95.9)	
Single	11 (4.0)	1 (2.5)	16 (4.1)	
**Education level**				0.330
Cannot read and write	26 (6.1)	3 (7.5)	23 (6.0)	
Primary	141 (33.1)	8 (20.0)	133 (34.5)	
Secondary	171 (40.1)	19 (47.5)	152 (39.4)	
Tertiary	88 (20.7)	10 (25.0)	78 (20.2)	
**Gestational age**^**d**^				< 0.001
37–39 weeks	231 (54.2)	12 (5.2)	219 (94.8)	
40–41 weeks	154 (36.2)	18 (11.7)	136 (88.3)	
> 41 weeks	41 (9.6)	10 (24.4)	31 (75.6)	
**Gravidity**				0.004
1	218 (51.2)	11 (27.5)	207 (53.6)	
2–4	151 (35.5)	23 (57.5)	128 (33.2)	
≥ 5	57 (13.4)	6 (15.0)	51 (13.2)	
**Anaemia during pregnancy**	5 (1.2)	5 (5.0)	3 (0.8)	0.072
**History of hypertension**	6 (1.4)	0 (0)	6 (1.6)	1.000
**HIV in pregnancy**	47 (11.0)	2 (5.0)	45 (11.7)	0.289
**Had malaria in pregnancy**	19 (4.5)	6 (15.0)	13 (3.4)	0.001
**Booking blood pressure**				0.525
< 130/80 mmHg	288 (67.6)	25 (62.5)	263 (68.1)	
≥ 130/80 mmHg	138 (32.4)	15 (37.5)	123 (31.9)	
**Body mass index (BMI) **^**b**^				0.007
> 25 kg/m^2^	379 (89.0)	30 (75.0)	349 (90.4)	
≤ 25 kg/m^2^	47 (11.0)	10 (25.0)	37 (9.6)	
**Estimated fetal weight** ^c^				0.181
< 2.5 kg	30 (7.0)	5 (12.5)	25 (6.5)	
2.5–3.5 kg	257 (60.3)	26 (65.0)	231 (59.8)	
> 3.5 kg	139 (32.6)	9 (22.5)	130 (33.8)	
**Low lying placenta**	11 (2.6)	39 (97.5)	376 (97.4)	0.973

*BMI* Body mass index

^a^ mean age = 26 (± 5) years

^b^ mean BMI = 28.9 (± 4.0) kg/m^2^

^c^ Mean fetal weight = 3.3 (± 0.5) kg

^d^ Mean gestational age = 39 (± 1.5) weeks

### Prevalence of oligohydramnios

Of the 426 participants who underwent sonographic assessment for AFI, 40 had AFI ≤ 5 cm, giving a prevalence of oligohydramnios of 9.4% (95%CI: 6.8–12.6%).

### Factors associated with oligohydramnios

In the multivariable analysis (Table 2), the odds of oligohydramnios were 2.5 times higher among participants with a history of malaria in pregnancy, compared to those who had no malaria in pregnancy. Prime gravidas were 3.7 times more likely to be diagnosed with oligohydramnios compared to multigravidas. There was an association between increasing gestational age and oligohydramnios; compared to mothers at 37–39 weeks, the odds of oligohydramnios were 2.5 times higher among participants who were at 40–41 weeks, and 6 times higher among participants who were at > 41 weeks of gestation.

**Table 2:** Factors associated with oligohydramnios among women at 37 weeks of gestation or beyond attending Mbarara Regional Referral Hospital, southwestern Uganda, November 2019–March 2020

**Characteristic**	**%Oligoydramnios**	**Unadjusted Analysis**		**Adjusted Analysis**	
	**n/N (%)**	**OR (95%CI)**	***P* value**	**Adjusted OR (95%CI)**	***P* value**
**Age category in years**					
< 25	15/156 (9.6)	Ref		Ref	
≥ 25	25/270 (9.3)	0.96 (0.49—1.9)	0.903	1.7 (0.76—3.9)	0.196
**Anaemia in pregnancy**					
No	38/421 (9.0)	Ref		Ref	
Yes	2/5 (40.0)	6.72 (1.1—42)	0.040	4.5 (0.57—36)	0.154
**Had malaria in pregnancy**					
No	34/407 (8.4)	Ref		Ref	
Yes	6/19 (31.6)	5.1 (1.8—14)	0.002	4.6 (1.5—14)	0.008
**Gravidity**					
1	23/151 (15.2)	3.4 (1.6—7.2)	0.001	3.7 (1.6—6.7)	0.002
2–4	11/218 (5.1)	Ref		Ref	
≥ 5	6/57 (10.5)	2.2 (0.78—6.3)		2.6 (0.85—7.8)	0.094
**Gestational age**					
37—39 weeks	12/231 (5.2)	Ref		Ref	
40—41 weeks	18/154 (11.7)	2.4 (1.1—5.2)	0.023	2.5 (1.1 – 5.6)	0.022
> 41 weeks	10/41 (24.4)	5.9 (2.4—15)	< 0.001	6.0 (2.3—16)	< 0.001
**Booking blood pressure in mmHg**					
< 130/80	25/288 (8.7)	Ref			
≥ 130/80	15/138 (10.9)	1.28 (0.65—2.52)	0.469		
**Body mass index in kg/m2**					
> 25 kg/m2	30/379 (7.9)	Ref		Ref	
≤ 25 kg/m2	10/47 (21.3)	3.1 (1.4—6.9)	0.005	2.88 (0.98—7.5)	0.053
**Estimated fetal weight in kilograms**					
< 2.5	1/14 (7.1)	Ref			
2.5—3.5	28/265(10.6)	1.54 (0.19—12)	0.685		
> 3.5	11/148 (7.5)	1.1 (0.13—8.8)	0.963		

*Ref* Reference category, *OR* Odds ratio, *CI* Confidence interval

## Discussion

In this hospital-based cross-sectional study in southwestern Uganda, we detected oligohydramnios in about one of every ten women at 37 weeks of gestation or beyond. Oligohydramnios was more common among participants with a history of malaria in pregnancy, those carrying their first pregnancy, and those with higher gestational age (40 weeks or beyond). Overall, these findings support the need to strengthen oligohydramnios surveillance in resource-constrained settings to enhance detection of this complication.

The prevalence of oligohydramnios of 9.4% reported in this study is similar to the prevalence of 11% reported in Italy [17] in a hospital setting. However, it is higher than the prevalence of 4.4% that was reported in China [18]. Much higher prevalence estimates compared to ours have been reported previously in India (17%) and South Africa (23%) [19, 20]. The variation of the previous prevalence estimates from ours may be attributed to the differences in methodologies employed across the different studies. For instance, the study in China audited deliveries in various hospitals and relied on secondary data from individual medical records, while ours relied largely on primary data to ascertain the presence or absence of oligohydramnios. Unlike our study, the study in India did not exclude women with ruptured membranes, while Buchmann and colleagues in South Africa considered only mothers referred due to post-term pregnancies. This may explain the much higher prevalence rates reported in these two studies.

Of note, mothers with a history of malaria in the current pregnancy had significantly higher odds of oligohydramnios compared to those who did not have malaria in pregnancy. This is a new finding. A study conducted in Brazil among pregnant women with vivax malaria reported no association of malaria and oligohydramnios [21]. However, it is worth noting that in the same study in Brazil, sonographic assessment done during the period of infection revealed thickened placentas with mal perfusion of the placentas, which may be a predisposing factor for oligohydramnios later in the pregnancy. In our study, we considered a record of documented diagnosis of malaria. There is also evidence of persistence of proteins that encode for apoptosis after placental malaria has been cleared by pharmaceutical agents [22]. This study also considered vivax malaria, which is not common in our setting in Uganda, and is less virulent compared to *Plasmodium falciparum,* that has been found to be more common in our setting [23]*.* Another study in Malawi reported adverse birth outcomes due to sequestration of *Plasmodium falciparum* parasites in the placenta [24]. Furthermore, malaria also leads to a dysregulation of angiopoietin, a protein that is responsible for vascular development in the placenta [25]. In cases of placental insufficiency and chronic hypoxia, the fetus adapts to the new situation to protect its important organs, by redistributing the blood flow. This mechanism leads to decreased diuresis and secretion of pulmonary fluids and subsequently leads to oligohydramnios [26]. These underlying pathological mechanisms may explain the association between oligohydramnios and malaria. Nevertheless, future studies in our Ugandan setting with *Plasmodium falciparum* endemicity are warranted to corroborate our research findings.

In agreement with previous findings [27], we found an association between increasing gestational age and oligohydramnios. Some studies have reported that in post-term pregnancies, the majority (93%) of oligohydramnios cases are idiopathic [28] and about 7% of the cases have placental insufficiency [26]. In post term pregnancies, alteration in the expression of aquaporins (aquaporin-1 and aquaporin-3) on amnion, placenta and chorion are thought to be responsible for the reduction in amniotic fluid [29]. Furthermore, accelerated apoptosis or increased renal tubular reabsorption as a result of a more mature tubular system, have also been hypothesized as probable underlying mechanisms in pathogenesis of oligohydramnios in post-term pregnancies [30].

In agreement with previous studies [5, 31], the current study found that prime gravidas had higher odds of oligohydramnios compared to multigravidas. This may be because disorders of pregnancy are exaggerated in prime gravidas compared to multigravidas. Moreover, some complications, such as hyperemesis gravidarum, and infections (e.g., malaria), have been reported to be associated with oligohydramnios [5].

The following limitations should be considered when interpreting our findings. First, we are unable to draw causal inferences from the observed associations due to the cross-sectional nature of our study, that does not enable assigning time directionally between the exposures and the outcome. Second, due to lack of longitudinal outcome data, we were unable to assess the prognostic implications of oligohydramnios in our study population. This can be assessed in future longitudinal studies. Finally, this was a single-centre study in a regional referral hospital, hence our findings may not be generalizable beyond the population of pregnant women in similar peri-urban settings in Uganda. Despite these limitations, our study generated valuable epidemiological data, being one of the initial studies to estimate the burden and correlates of oligohydramnios in the East African Region.

## Conclusion

Oligohydramnios was detected in approximately one of every ten pregnancies beyond the gestational age of 36 weeks, in women seeking care at MRRH. Increasing gestational age, history of malaria in pregnancy, and prime gravidity were the factors significantly associated with oligohydramnios. We recommend increased surveillance for oligohydramnios in the third trimester, especially among prime gravidas, those with history of malaria in pregnancy, and those with post-term pregnancies, in order to enable prompt detection of this complication and plan timely interventions.

## Data Availability

The datasets generated and analysed are available from the corresponding author upon request.

## Abbreviations

ACEI: Angiotensin converting enzyme inhibitor
AFI: Amniotic Fluid Index
aOR: Adjusted Odds Ratio
BMI: Body Mass Index
CI: Confidence Interval
IQR: Interquartile Range
LNMP: Last normal menstruation period
MoH: Ministry of Health
MRRH: Mbarara Regional Referral Hospital
MUST: Mbarara University of Science and Technology
NSAID: Non-steroidal anti-inflammatory drug
OR: Odds Ratio
UTI: Urinary Tract Infection
SD: Standard Deviation
